# Hematopoietic Stem/Progenitor Cells Directly Contribute to Arteriosclerotic Progression via Integrin β_2_

**DOI:** 10.1002/stem.1939

**Published:** 2015-03-24

**Authors:** Xuhong Wang, Mingming Gao, Sarah Schouteden, Anton Roebroek, Kristel Eggermont, Paul P van Veldhoven, George Liu, Thorsten Peters, Karin Scharffetter-Kochanek, Catherine M Verfaillie, Yingmei Feng

**Affiliations:** a Beijing Key Laboratory of Diabetes Prevention and Research, Department of Endocrinology, Lu He Hospital, Capital Medical UniversityBeijing, People's Republic of China; b Institute of Cardiovascular Sciences, Peking UniversityBeijing, People's Republic of China; c Interdepartmental Stem Cell Institute, Katholieke Universiteit LeuvenBelgium; d Department of Human Genetics, Katholieke Universiteit LeuvenBelgium; e Laboratory of Lipid Biochemistry and Protein Interactions, Department of Cellular and Molecular Medicine, Katholieke Universiteit LeuvenLeuven, Belgium; f Department of Dermatology and Allergic Diseases, Ulm UniversityUlm, Germany

**Keywords:** Hematopoietic stem/progenitor cells, Low-density lipoprotein, Arteriosclerosis, Extracellular regulated protein kinase, Integrin, Inflammation

## Abstract

Recent studies described the association between hematopoietic stem/progenitor cell (HSPC) expansion in the bone marrow (BM), leukocytosis in the peripheral blood, and accelerated atherosclerosis. We hypothesized that circulating HSPC may home to inflamed vessels, where they might contribute to inflammation and neointima formation. We demonstrated that Lin^−^ Sca-1^+^ cKit^+^ (LSK cells) in BM and peripheral blood of LDLr^−/−^ mice on high fat diet expressed significantly more integrin β_2_, which was responsible for LSK cell adhesion and migration toward ICAM-1 in vitro, and homing to injured arteries in vivo, all of which were blocked with an anti-CD18 blocking antibody. When homed LSK cells were isolated from ligated artery and injected to irradiated recipients, they resulted in BM reconstitution. Injection of CD18^+/+^ LSK cells to immunodeficient Balb/C Rag2^−^ γC^−/−^ recipients resulted in more severe inflammation and reinforced neointima formation in the ligated carotid artery, compared to mice injected with PBS and CD18^−/−^ LSK cells. Hypercholesterolemia stimulated ERK phosphorylation (pERK) in LSK cells of LDLr^−/−^ mice in vivo. Blockade of pERK reduced ARF1 expression, leading to decreased integrin β_2_ function on HSPC. In addition, integrin β_2_ function could be regulated via ERK-independent LRP1 pathway. Integrin β_2_ expression on HSPC is regulated by hypercholesterolemia, specifically LDL, in pERK-dependent and -independent manners, leading to increased homing and localization of HSPC to injured arteries, which is highly correlated with arteriosclerosis. Stem Cells *2015;33:1230–1240*

## Introduction

Inflammatory cells in atherosclerotic plaques are exclusively derived from hematopoietic stem/progenitor cells (HSPC). Recently, others and we reported that hypercholesterolemia promotes HSPC proliferation and differentiation toward atherogenic monocytes and granulocytes; of which elevated peripheral white blood cell levels have been described in atherosclerotic patients [1–3]. Although the link among HSPC proliferation in bone marrow (BM), leukocytosis in peripheral blood (PB), and accelerated atherosclerosis progression was noted, there is to date no evidence demonstrating the direct involvement of HSPC in plaque development.

It is well-known that HSPC reside in the hypoxic BM niche and give rise to all blood cells in postnatal life [4]. Under steady state conditions, a fraction of HSPC can be found in PB [5]. Circulating HSPC can migrate into peripheral tissues such as spleen, liver, lymph node, and aortic adventitia [6–9]. Although circulating HSPC migrate into lymph nodes and proliferate and differentiate in situ to become resident myeloid cells for host defense [6], the fate of HSPC that traffic into damaged tissues remains largely unknown. Nevertheless, there is evidence that when CD34+ hematopoietic progenitor cells migrate into the injured spinal cord, exacerbated neuroinflammation and progression of multiple sclerosis are observed [10]. Likewise presence of HSPC in asthmatic airways is associated with asthma progression [11]. HSPC trafficking in injured liver, by contrast, appears to be associated with improved liver function, even if the mechanism is unknown [8],[9]. We here tested the hypothesis that HSPC, under the influence of hypercholesterolemia, can home into the intima of inflamed arteries and contribute to arteriosclerotic plaques formation.

HSPC self-renewal versus differentiation and retention of HSPC within the BM niche are regulated by HSPC intrinsic factors as well as by extrinsic factors via engagement of cytokine and growth factor receptors and adhesion receptors by their specific ligands. The integrins including α4β1, α5β1, β2, α6 and some chemokines such as C-X-C chemokine receptor type 4 (CXCR4) and CD192 (i.e. CCR2) play important roles in retention and homing of HSPC from blood to the BM niche [12–15], mobilization of HSPC from the BM into the PB [16–19], and trafficking into peripheral tissues [8–10,20]. In addition, CXCR4 has been identified as a key factor in hypercholesterolemia-induced HSPC mobilization from BM into PB [17,21,22]. However, it remains unknown what other key adhesion molecules are also affected and thus modify HSPC function by hypercholesterolemia. Expression and function of these adhesion receptors are regulated by a variety of signaling cascades, including the mitogen activated protein kinases (MAPK) signaling pathway [23–25]. Recently, Yves-Charvet reported extracellular signal regulated kinases 1/2(ERK1/2) activation in HSPC from hypercholesterolemic *Abca1^−^^/^^−^ Abcg1^−^^/^^−^* mice [3]. In line with this study, our data demonstrated that low-density lipoprotein (LDL)-mediated differentiation of HSPC to granulocytes occurs in response to LDL-stimulated ERK1/2 activation [2]. This led us to determine if LDL affects integrin function and hence migration of HSPC into arteriosclerotic plaques via activation of the ERK pathway.

LDL receptor-related protein (LRP) is a member of the LDL-receptor family. It is expressed in a variety of cell types such as hepatocytes and leukocytes. More than 30 ligands have been discovered which explains the multiple functions of LRP [26]. Like other members in this family, LRP1 mediates cholesterol uptake via endocytosis. Aside from its function in cholesterol homeostasis, LRP1 has been found to interact with integrin β2 in leukocytes and therefore modulate integrin clustering on the membrane [27]. LRP1 deficiency abrogated integrin β2-dependent adhesion of leukocytes to endothelial cells [28]. Interestingly, an intimate association between LRP1 expression and ERK phosphorylation has been noticed in different cell types, all of which modulate cell adhesion and migration [29–31]. However, it is currently unknown if LRP1 regulates HSPC adhesion, migration or homing.

Here we report that hypercholesterolemia increased the percentage of integrin β_2_^+^ Lin^−^ Sca-1^+^ cKit^+^ (LSK) cells in LDLr*^−^*^/^*^−^* mice. Integrin β_2_ regulated LSK cell adhesion and migration toward to ICAM and homing to injured artery. Grafted integrin β_2_^+/+^ LSK cells resulted in enhanced inflammation and neointima formation in the ligated artery, compared to injection of PBS and integrin β_2_*^−^*^/^*^−^* LSK cells. Finally, we demonstrate that LDL effects on integrin β_2_ expression and function are mediated by the ERK/ADP-ribosylation factor 1 (ARF1)-dependent and ERK-independent LRP1 pathway.

## Materials and Methods

Integrin β_2_ expressing HSPC were studied in LDLr*^−^*^/^*^−^* mice fed on chow or high fat diet (HFD) (34% fat, 1% cholesterol, Catalog no. D12492 mod, BioServices, The Netherlands, http://www.researchdiets.com/collection1?q=D12492). Complete ligation of right carotid artery was performed on B.6SJL-PTPRCA (CD45.1) mice, wild type (WT) C57BL/6J (CD45.2, H-2kb) mice, CD18^−/−^ mice and their littermates or Balb/c Rag2− γC^−/−^ mice (H-2kd) mice for HSPC homing and injection experiment. Detailed methods are shown in Supporting Information data.

## Results

### Hypercholesterolemia Increased Integrin β_2_ Expression on LSK Cells

**Figure 1:**
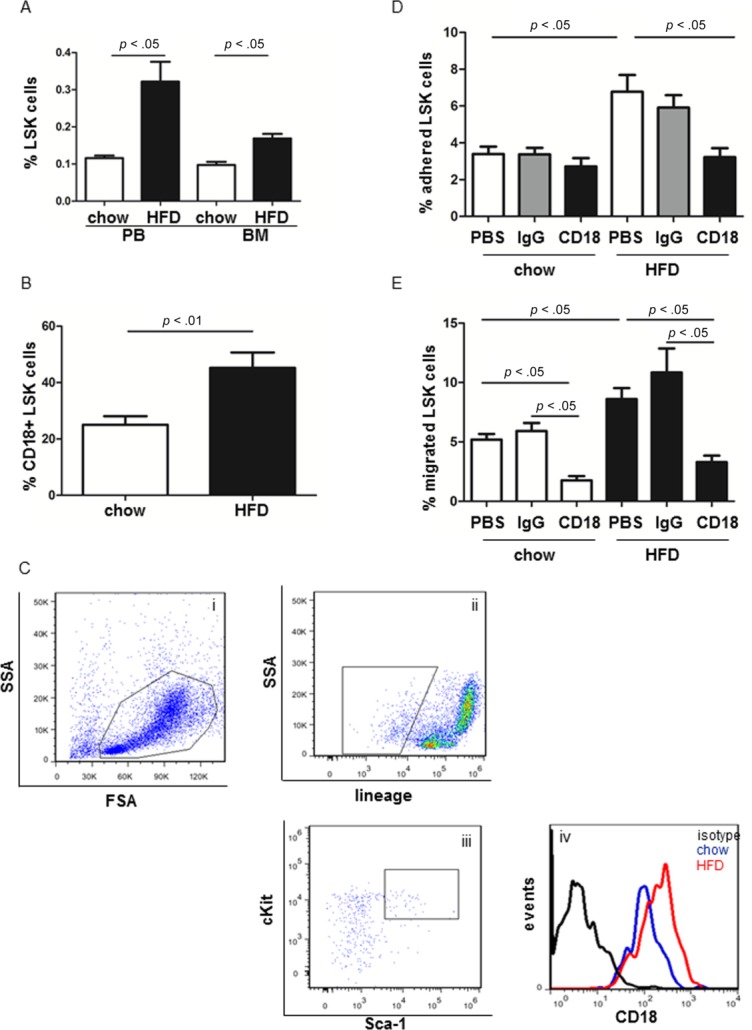
In vivo hypercholesterolemia increases integrin β_2_ expression on LSK cells. LDLr^−/−^ mice were fed on chow or HFD for 8 weeks. (A): Frequency of LSK cells in PB and BM cells (BMC). (B): The percentage of integrin β_2_^+^ LSK cells in the LSK population. (C): Representative fluorescence-activated cell sorting (FACS) analysis of integrin β_2_^+^ LSK cells: (a) living BMC were shown in the box; (b) Lin− cells when gated on living BMC; (c) LSK cells when gated on Lin− cells; (d) integrin β_2_ expressing LSK cells when gated on LSK population. (D): To evaluate the adhesion capacity of LSK cells from mice on HFD vs. chow diet, LSK cells were isolated by FACS and allowed to adhere to ICAM-1-coated plates. After 2 hours, adherent LSK cells were detached with 0.25% trypsin and counted by FACS. *n* = 5–13. (E): Lin− cells isolated from chow and HFD mice were plated in transwells coated with ICAM-1 for 6 hours. The percentage of LSK cells that migrated through the transwells was assessed. *n* = 5–11. Abbreviations: BM, bone marrow; FSA, forward scatter area; HFD, high fat diet; LSK, Lin^−^ Sca-1^+^ cKit^+^; PB, peripheral blood; PBS, phosphate buffered saline; SSA, side scatter area.

Adhesion molecules play critical roles in LSK function. Therefore, we first screened integrin expression on HSPC in LDLr^−/−^ mice on chow diet and HFD. After 8 weeks of HFD, total cholesterol, LDL-c and high-density lipoprotein cholesterol (HDL-c) were dramatically increased in LDLr^−/−^ mice compared to mice on chow diet (Supporting Information Fig. 1). Consistent with our previous findings [2], the frequency of LSK cells was significantly increased in PB and BM of LDLr^−/−^ mice on HFD compared to those on chow diet (PB: 0.32% ± 0.053% vs. 0.12% ± 0.007%, *n* = 8–10, *p* < .01; BM: 0.17% ± 0.012% vs. 0.10% ± 0.008%, *n* = 8–10, *p* < .01) (Fig. 1A). When BM cells (BMCs) were stained with anti-CD18 and LSK Abs, the percentage of CD18^+^ LSK cells in the LSK population from mice on HFD was higher than that of the chow diet group (%CD18^+^ LSK cells: Chow: 25.04% ± 3.02%; HFD: 45.25% ± 5.41%, *n* = 7–8, *p* < .01) (Fig. 1B, 1C). Integrin β1 and α5 expression was not different between LDLr^−/−^ mice on chow and HFD (data not shown).

To determine if β_2_ on LSK cells was functional, LSK cells from LDLr^−/−^ mice on chow and HFD were isolated by fluorescence-activated cell sorting (FACS), incubated with or without a blocking anti-CD18 Ab or IgG control Ab for 20 minutes prior to the adhesion assay. LSK cells were plated on intercellular adhesion molecular-1 (ICAM-1)-coated 96-well plate for 2 hours. After nonadherent LSK cells were removed, adherent LSK cells were mixed with beads and enumerated by FACS. LSK cells from LDLr^−/−^ mice on HFD displayed a 2.6-fold greater adhesion capacity to ICAM-1 compared with those from mice on chow diet, which was inhibited by a blocking anti-CD18 Ab (*n* = 5–8, Fig. 1D).

To further evaluate whether increased integrin β2 expression was associated with enhanced LSK migration over ICAM-1, Lin− cells were isolated from LDLr^−/−^ mice on chow and HFD, treated with phosphate buffered saline (PBS), anti-CD18 or IgG control Abs for 20 minutes, and then plated in transwells coated with ICAM-1. After 6 hours, cells in the lower chamber were collected, counted, and stained with anti-LSK Abs. The input Lin− population was also stained with LSK Abs to enumerate the absolute number of LSK cells that had migrated to the lower chamber. LSK cells from HFD mice displayed a 1.6-fold higher migration capacity through ICAM-1-coated transwells compared to those from mice on chow diet. Anti-CD18 Abs inhibited LSK migration from mice on chow diet and HFD (*n* = 5–11, Fig. 1E).

### HSPC Could Home to Inflamed Arteries via Integrin β2

**Figure 2:**
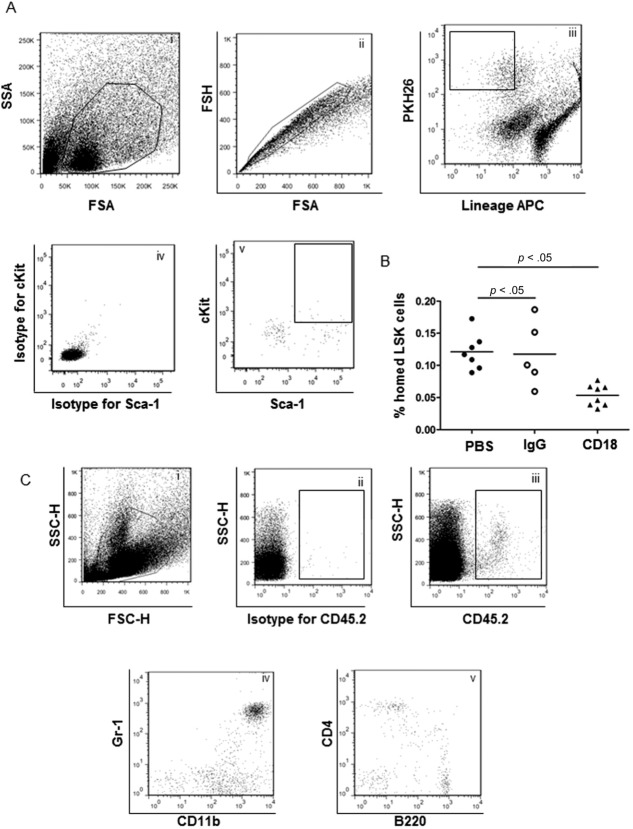
Hematopoietic stem/progenitor cell (HSPC) could home to lesion site via integrin β_2_. (A): Lin− cells from LDLr^−/−^ mice on high fat diet were labeled with PKH26 and then injected by tail vein in mice that had undergone carotid artery ligation. The fraction of homed LSK cells was identified by fluorescence-activated cell sorting (FACS). (i) Living artery cells shown in the box; (ii) single artery cell suspension; (iii) PKH26+ Lin− cells were shown when gated on single cells; when compared to the isotype (iv), homed LSK cells were identified (v). (B): Quantification of homed LSK cells in recipients that were injected with PBS, Lin− cells treated with IgG Ab or CD18 Ab. (C): Homed PKH26+/LSK cells were sorted out by FACS and injected in irradiated CD45.1 mice via tail vein. After 16 weeks, donor-derived myeloid and lymphoid cells were identified by FACS. (i) Living bone marrow cell shown in the box; compared with cells stained with isotype IgG PerCP-Cy5.5 (ii), CD45.2+ cells were identified (iii); (iv) CD45.2+ derived granulocytes and monocytes; (v) CD45.2-derived B cells and T cells. Abbreviations: APC, allophycocyanin; FSA, forward scatter area; FSH, forward scatter height; LSK, Lin^−^ Sca-1^+^ cKit^+^; PBS, phosphate buffered saline; SSA, side scatter area; SSC, side scatter.

We next assessed the role of integrin β_2_ on HSPC homing to injured arteries in vivo. Ligation of carotid artery has been shown to induce ICAM-1 expression on injured endothelial cells [32,33]. Therefore, this model was used in the entire study. Lin− cells isolated from LDLr^−/−^ mice on HFD were labeled with PKH26 and then injected intravenously into mice wherein the carotid artery was ligated 3 days earlier. Prior to injection, cells were incubated with PBS, IgG Ab, or anti-CD18 Ab for 20 minutes. The following day, the injured and noninjured carotid arteries were dissected after extensive perfusion to remove blood cells in the arteries, digested, and stained with anti-LSK Abs. FACS data demonstrated that homing of LSK cells to injured artery was blocked by CD18 antibody (% homed LSK cells: PBS group: 0.120% ± 0.0107%; IgG group: 0.118% ± 0.0228%; CD18 group: 0.053 ± 0.0059; *n* = 5–8) (Fig. 2A, 2B).

To explore the kinetics of homed LSK cells to injured arteries, carotid artery ligation was performed on CD45.1 mice and then they were received Lin− cells isolated from LDLr^−/−^ mice on HFD. One and ten days after cell injection, CD45.1 mice were sacrificed, and both injured and uninjured carotid arteries were dissected. After digestion, artery cells were numerated and stained with anti-mouse CD45.2 and LSK markers for FACS. The absolute number of homed LSK cells in uninjured and injured arteries was calculated. The number of donor-derived LSK cells was higher in injured arteries compared to uninjured arteries at both day 1 and day 10 after injection (day 1: 34 ± 10.3 cells per graft vs. 308 ± 63.6 cells per graft, *n* = 7, *p* < .01; day 10: 41 ± 10.1 cells per graft vs. 219 ± 34.5 cells per graft, *n* = 6, *p* < 0.01) (Supporting Information Fig. 2).

To prove that LSK cells homed to the injured arteries were HSPC, PKH26+ LSK cells were isolated by FACS and injected together with CD45.1 BMCs to irradiated CD45.1 recipients. After 16 weeks of BM transplantation, recipient blood cells were stained with anti-CD45.1 FITC, anti-CD45.2 PerCP-Cy5.5 together with myeloid and lymphoid markers for chimerism analysis. CD45.2-derived granulocytes, monocytes, B cells, and T cells were identified by FACS (*n* = 5, Fig. 2C). These data indicate that homed PKH26^+^ LSK cells are indeed HSPC.

### CD18 Deficiency Was Associated with Leukocytosis But No Difference in HSPC Frequency in BM

**Figure 3:**
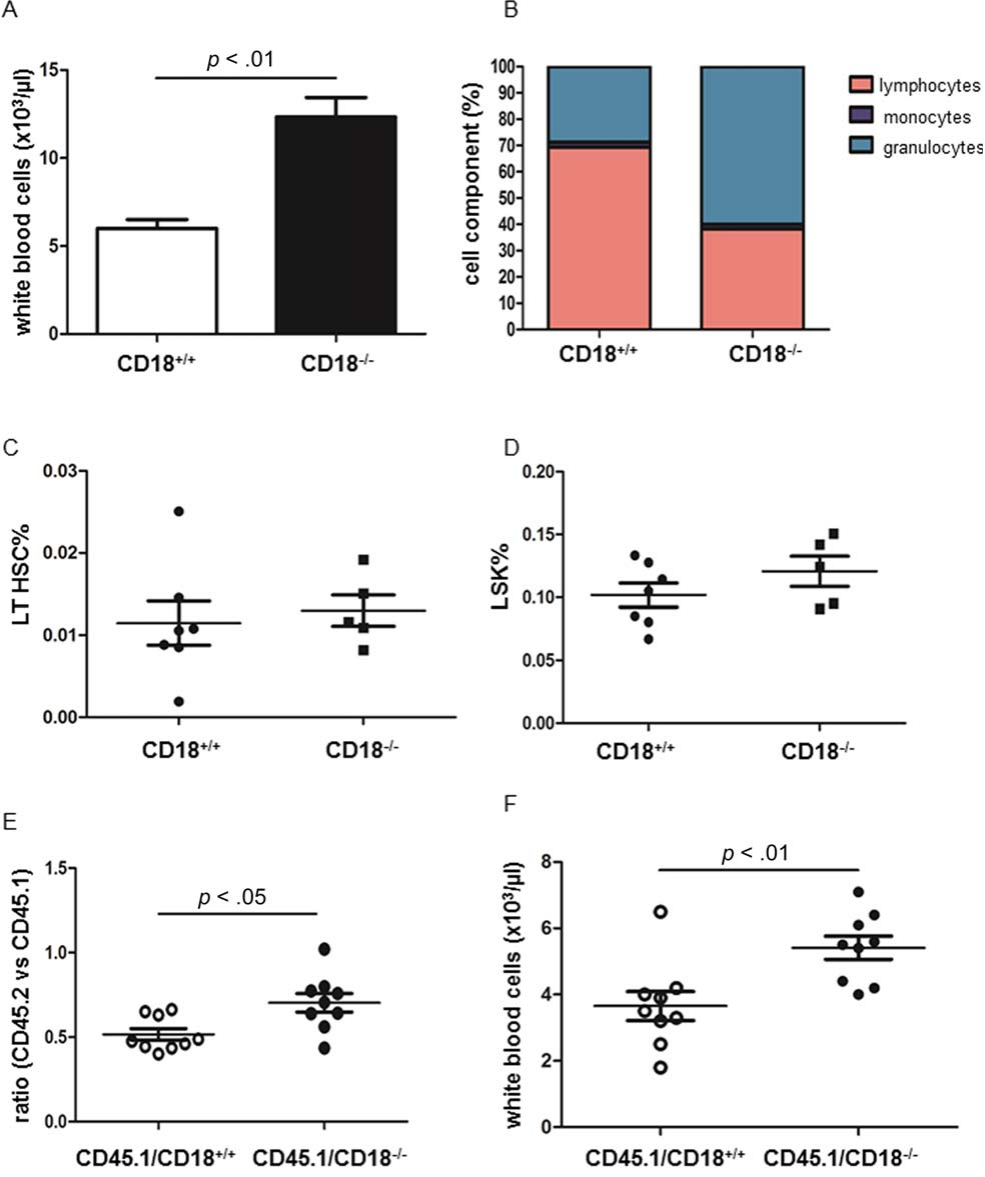
Phenotypic characterization of CD18^−/−^ hematopoietic stem/progenitor cell. (A): WBC count of CD18^+/+^ and CD18^−/−^ mice. (B): The percentage of lymphocytes, monocytes, and granulocytes in white blood cell (WBC) of CD18^+/+^ and CD18^−/−^ mice. (C, D): The frequency of LT HSC and LSK cells LSK frequency in bone marrow (BM). (E): CD18^+/+^ or CD18^−/−^ BM cell (BMC) were mixed with equal amount of CD45.1 BMC before transplanted to irradiated CD45.1 recipients. Eight weeks after transplantation, peripheral blood of recipients was stained with CD45.1 and CD45.2 for chimerism (E). The WBC count of CD45.1 recipients was shown 8 weeks after BM transplantation. Abbreviations: LSK, Lin^−^ Sca-1^+^ cKit^+^; LT HSC, long-term hematopoietic stem cell.

To further investigate the role of CD18^−/−^ HSPC in grafting and arterosclerosis development, CD18^−/−^ mice were generated as described before [34]. CD18^−/−^ mice displayed dramatic increased white blood cells in PB with skewed granulocyte production (Fig. 3A, 3B). Despite leukocytosis, percentage of long-term HSC and LSK in BMC were not differed between CD18^+/+^ and CD18^−/−^ mice (Fig. 3C, 3D). When equal amount of CD18^+/+^ BMC or CD18^−/−^ BMC were competitively transplanted to irradiated CD45.1 recipients, transplantation of CD18^−/−^ BMC resulted in more white blood cells (WBC) count in the recipients (Fig. 3E, 3F). Despite greater amount of WBC in CD18^−/−^ mice, when carotid artery ligation was performed in CD18^−/−^ mice and their littermates, neointima formation was not differed between two groups after 28 days of surgery (neointima area: 41,770 ± 12,207.0 µm^2^ vs. 41,814 ± 9,322.8 µm^2^, *n* = 4–5).

### Injection of CD18^+/^^+^ LSK Cells Resulted in More Neointima Formation than CD18^−^^/^^−^LSK Cells

**Figure 4:**
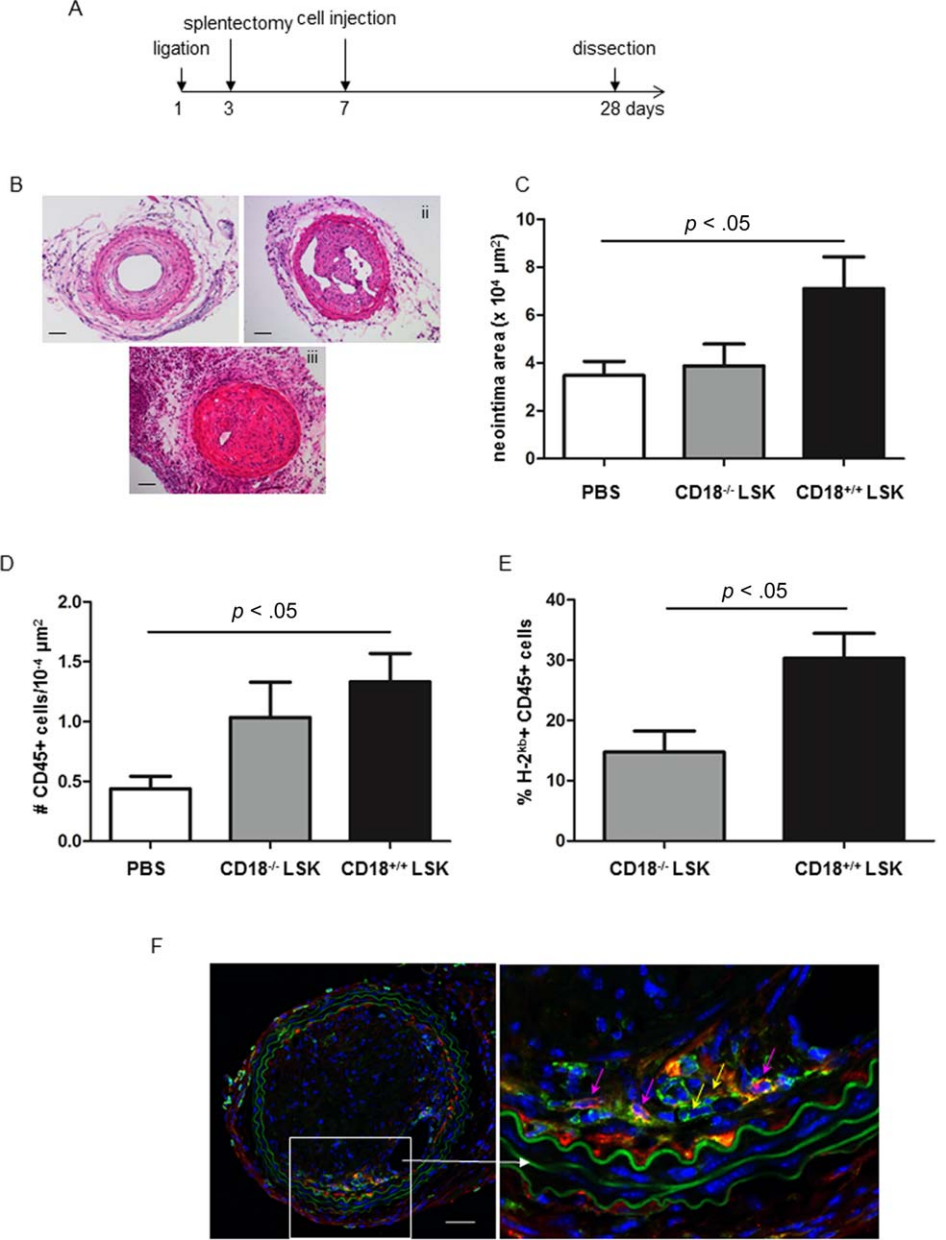
Hematopoietic stem/progenitor cell injection reinforced inflammation and accelerated neointima formation in mice that underwent carotid artery ligation. (A): The scheme of the cell injection experiment. (B): Representative H&E sections of carotid arteries of Balb/C Rag2− γC^−/−^ mice injected with PBS (i), CD18^−/−^ LSK cells (ii), and CD18^+/+^ LSK cells (iii). Scale bar = 50 µm. (C): Neointima area in the ligated artery of Balb/c Rag2− γC^−/−^ mice received saline, CD18^+/+^ LSK, and CD18^−/−^ LSK cells. *n* = 4–7 per group. (D): Cryosections were stained with rat anti-mouse CD45 and mouse anti-mouse H-2kb Abs, and then goat anti-rat Alexa 488 and goat anti-mouse Alexa 555 Abs. CD45+ cell density was obtained by the number of CD45+ cells divided by neointima area. (E): The percentage of donor-derived CD45+ cells in the lesion site. (F): Identification of donor-derived CD45+ cells. Yellow arrows indicate CD45+ cells derived from recipients, whereas pink arrows indicate CD45+ cells originated from donor HSPC. Scale bar = 100 µm. Abbreviations: LSK, Lin^−^ Sca-1^+^ cKit^+^; PBS, phosphate buffered saline.

To assess the fate of HSPC homing to injured artery, we grafted HSPC to immunodeficient Balb/C Rag2^−^ γC^−/−^ recipients (H-2kd) that underwent a complete ligation of the right carotid artery. Three days after ligation, splenectomy was performed to improve HSPC homing (Fig. 4A). Seven days after artery ligation, 40,000 LSK cells isolated from CD18^+/+^ or CD18^−/−^ mice were injected to Balb/C Rag2− γC^−/−^ recipients. Four weeks after ligation surgery, recipients were sacrificed and the histology of the plaques was examined. H&E analysis showed that administration of CD18^+/+^ LSK cells resulted in greater neointima formation compared to mice injected with PBS or CD18^−/−^ LSK cells (n=4–7, Fig. 4B, 4C). Cryosections were stained with antibodies against H-2kb and CD45 and inflammation index was assessed. Quantification of CD45+ cells illustrated that mice that were injected with CD18^+/+^ LSK cells developed aggravated neointima inflammation compared to mice received PBS and CD18^−/−^ LSK cells (Fig. 4D). In addition, the percentage of H-2kb+ CD45+ cells among CD45+ cells was twofold higher in mice injected with CD18^+/+^ LSK cells compared to mice received CD18^−/−^ LSK cells (Fig. 4E). Identification of donor-derived CD45+ cells, that is, H-2kb^+^ CD45^+^ cells was illustrated in Figure 4F.

### ERK1/2 Phosphorylation Is Involved in LDL-Mediated Integrin β2 Induction on HSPC

**Figure 5:**
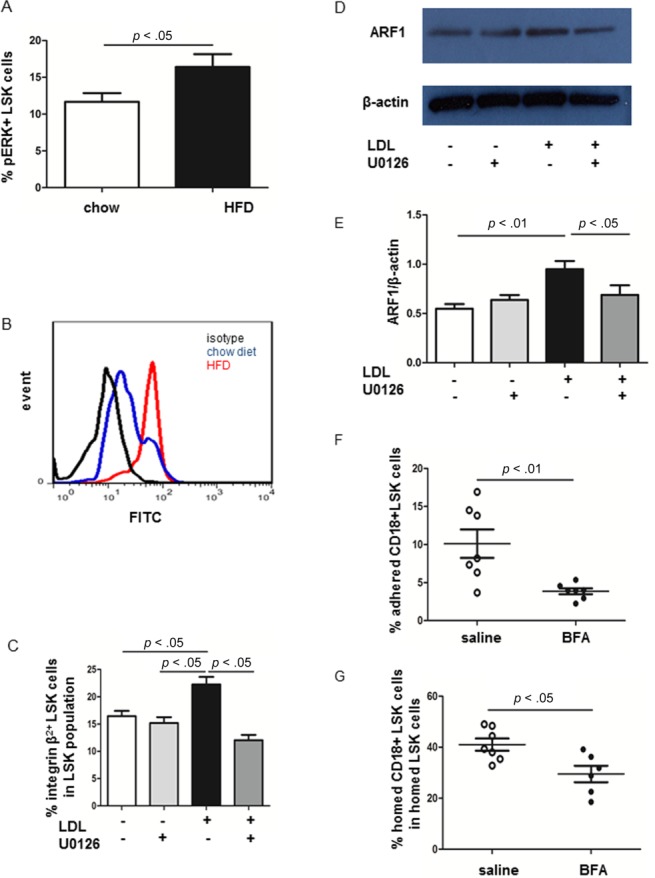
Role of pERK on integrin β_2_ expression. (A): Quantification of pERK+ LSK cells in LDLr^−/−^ mice on chow or HFD. (B): Fluorescence-activated cell sorting analysis of pERK+ LSK cells. (C): Lin− cells were exposed to 0 or 100 µg/ml LDL with or without 10 µM U0126 for 24 hours. Cells were stained with Abs against LSK cells and anti-CD18 for FACS. The percentage of integrin β_2_^+^ LSK cells in the LSK population; *n* = 5–6. (D): Representative Western blot of ARF1 and β-actin expression in Lin− cells that were treated with 0 or 100 µg/ml LDL in the presence or absence of 10 µM U0126 for 24 hours. (E): Quantification of ARF1 protein expression normalized with β-actin. *n* = 6. (F): Lin− cells were exposed to 0 or 5 µM BFA overnight. Equal amounts of cells were plated into ICAM-1-coated plates and allowed to adhere for 2 hours. Data are expressed as the percentage of adhered integrin β_2_^+^ LSK cells divided by the total number of seeded integrin β_2_^+^ LSK cells. *n* = 7. (G): Lin− cells were treated with or without BFA as described above and labeled with PKH26 before injection into ligated CD45.2 recipients. The percentage of integrin β_2_^+^ LSK cells within the LSK cell population was quantified. *n* = 6–7. Abbreviations: ARF1, ADP-ribosylation factor 1; BFA, Brefeldin A; FITC, fluorescein; HFD, high fat diet; LDL, low density lipoprotein; LSK, Lin^−^ Sca-1^+^ cKit^+^; pERK, phospho ERK.

Thereafter, to gain insight in how hypercholesterolemia induces β_2_ expression, we first stained BMC harvested from LDLr^−/−^ mice on chow and HFD with anti-pERK (p42/p44) and LSK Abs as described before [2]. Consistent with our previous in vitro findings [2], we found a 1.4-fold increase in the percentage of pERK^+^ LSK cells in LDLr^−/−^ mice on HFD compared with chow diet (% pERK^+^ LSK in total LSK cells: chow diet: 11.7% ± 1.18%; HFD: 16.4% ± 1.74%, *n* = 8–9, *p* < .05) (Fig. 5A, 5B).

We established in vitro assays to explore the effects of lipoproteins on LSK cells. Lin− cells were isolated from LDLr^−/−^ mice and exposed to equal amount of LDL or HDL (0–100 µg/ml) for 24 hours. Cells were harvested and stained with anti-mouse CD18 and LSK markers for FACS as described above. The percentage of CD18+ LSK cells in LSK population was 1.4-fold higher at 25 µg/ml LDL, 1.5-fold higher at 50 µg/ml LDL, and 1.6-fold higher at 100 µg/ml LDL than non-LDL treated cells. Distinct from LDL, HDL treatment did not increase CD18 expressing LSK cells in vitro (Supporting Information Fig. 3).

We next assessed if pERK is involved in the induction of β_2_ integrin expression by LDL. Lin− cells were cultured with 100 µg/ml LDL in the presence or absence of pERK inhibitor, U0126, for 24 hours. Cells were stained with Abs against LSK and integrin β_2_. As was seen in vivo, LDL increased the fraction of LSK cells that expressed CD18 (*n* = 5–6, Fig. 5C). Culture of LSK cells with LDL in combination with U0126 attenuated these changes (Fig. 5C). By contrast, incubation with LDL did not alter the percentage of integrin α5^+^ LSK cells nor the percentage of integrin β_1_^+^ LSK cells (%integrin α5^+^ LSK cells: 89% ± 3.0% vs. 89% ± 1.8%; %integrin β_1_^+^ LSK cells: 96.7% ± 1.16% vs. 95.8 ± 1.74, *n* = 4).

Presentation of adhesion molecules on the cell membrane is a complex process which starts with gene transcription, translation, and transportation. The small G protein ARF1 has been shown to mediate receptor trafficking to the membrane, including the chemokine receptor CXCR4, integrin β1, and CD11a [35–38]. We thus evaluated whether LDL induced any change in ARF1 expression in Lin− cells. Western blot data illustrated that exposure of LDL to Lin− cells increased ARF1 expression in a pERK-dependent manner (Fig. 5D, 5E). To dissect whether ARF1 facilitated CD18 expression or function, Lin− cells were exposed to PBS or 5 µM Brefeldin A (BFA), an ARF1 inhibitor [36], for 8 hours and then stained with anti-CD18 and LSK Abs. BFA treatment neither altered the percentage of LSK cells in the population (control: 3.8% ± 0.44%; BFA: 3.5% ± 0.59%, *n* =7) nor led to significant change of CD18 expression on LSK cells. However, blocking ARF1 expression by BFA significantly reduced LSK cell adhesion to ICAM-1 in vitro and LSK cell homing to ligated arteries in vivo (*n* = 6–7, Fig. 5F, 5G).

### LRP1 is also in Part Responsible for LDL-Mediated Integrin β2 Induction on HSPC

**Figure 6:**
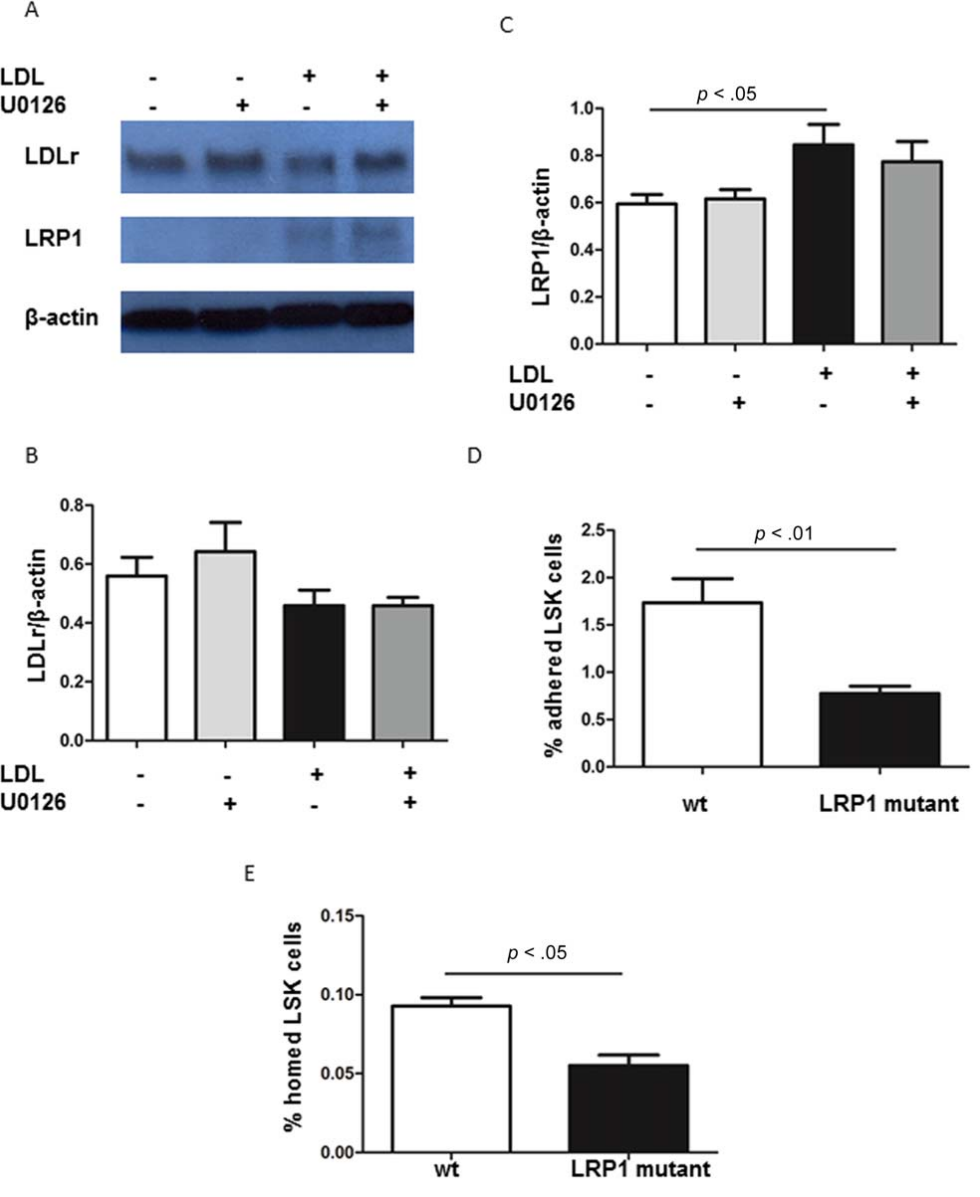
pERK independent regulation of integrin β_2_ expression on LSK cells. (A): Lin− cells from wild type mice were exposed to 0 or 100 µg/ml LDL with or without 10 µM U0126. Western blot was performed to study LDLr, LRP1, and β-actin expression. Representative Western blot of LDLr, LRP1, and β-actin were shown. (B, C): Quantification of the expression levels of LDLr and LRP1, normalized with β-actin in (B) and (C), respectively. *n* = 6. (D): LSK cells from LRP1 mutant versus wild type (WT) mice were selected by FACS and seeded onto ICAM-1 coated 96-well plates. Two hours later, adherent LSK cells were harvested and enumerated by FACS. Data are expressed as the percentage of adherent LSK cells in the input number of LSK cells. *n* = 5. (E): Lin− cells were isolated from LRP1 WT and mutant mice. After labeling with PKH26, cells were injected in mice with carotid artery ligation. Homed LSK cells to the ligated artery were quantified by FACS. *n* = 6–7. Abbreviations: LDL, low density lipoprotein; LRP1, LDL receptor-related protein 1; LSK, Lin^−^ Sca-1^+^ cKit^+^.

LRP1 belongs to the LDL receptor superfamily. LRP1, together with LDLr, regulate cholesterol homeostasis via endocytosis. Different from LDLr, LRP1 has multiple functions such as regulation of integrin expression and function [27,28,39]. We first determined if LRP1 is expressed on Lin− cells by Western blot analysis. LRP1 could not be detected in Lin− cells under control conditions but was induced by exposure of Lin− cells to LDL. By contrast, exposure to LDL did not significantly affect LDL receptor expression in Lin− cells (Fig. 6A–6C). Of note, inhibition of ERK by U0126 did not prevent induction of LRP1 expression in Lin− cells. To further assess the possible involvement of LRP1 in integrin-mediated HSPC homing to injured arteries, we isolated LSK cells from LRP1 mutant mice (LRP1^n2/n2^) [31] and repeated the function assays described above. LSK cells isolated from LRP1^n2/n2^ mice showed decreased adhesion to ICAM-1 in vitro and reduced homing to injured arteries in vivo, compared to WT LSK cells (*n* = 5–7, Fig. 6D, 6E). These data indicate that LRP1 is at least in part responsible for the regulation of integrin β2 function, while induction of LRP1 by LDL is ERK-independent.

## Discussion

Leukocytosis and monocytosis contribute substantially to arteriosclerotic progression [40–42]. Others and we have previously described that hypercholesterolemia induces HSPC proliferation and differentiation, resulting in expansion of the inflammatory cell pool in PB. However, it remains unclear if and how HSPC can migrate into lesion where they may locally expand and differentiate into leukocytes that further reinforce plaque formation. Till present, CXCR4 is the main factor identified to be responsible for HSPC mobilization in hypercholesterolemia. It would be very interesting to explore if other key factors that are modulated and in turn regulate HSPC function in the context of hypercholesterolemia. Therefore, we assessed if (a) HSPC directly contribute to arteriosclerosis progression; (b) hypercholesterolemia and LDL modulate migration, attachment, and homing of HSPC to injured artery, and if so by which mechanism.

Inflammation of blood vessels, which underlies the formation of atherosclerotic plaques, leads to upregulation of ICAM-1, the ligand for integrin β_2_. It is well-known that integrin β2 facilitates neutrophil and monocyte attachment and then transmigration through inflamed endothelium, which is why integrin β2 deficiency attenuates neointima formation in mice [43–45]. We demonstrate here that HSPC express significantly higher levels of CD18, when harvested from hypercholesterolemic mice, or following exposure of HSPC to LDL in vitro. We further demonstrate that homing of LSK to injured arteries occurs, which can be inhibited with blocking anti-CD18 antibodies. Grafting CD18^+/+^ LSK cells to recipients accelerated inflammation and arteriosclerosis in the injured artery, which is partially due to donor-derived inflammatory cells in the lesion. These data implicate the critical regulation of integrin β_2_ in HSPC adhesion, migration, and homing and further identify pro-atherogenic property of HSPC in arteriosclerosis development. Due to technical limitations, it is at present not feasible to trace HSPC proliferation and differentiation following homing to the lesion. Nevertheless, we postulate that both LSK proliferation and differentiation might occur in situ.

We previously demonstrated that pERK is responsible for LDL-mediated increased differentiation of HSPC in vitro. In this study, we reported that hypercholesterolemia is associated with pERK activation in LSK cells in vivo. We found that pERK is responsible for upregulation of the G protein ARF1, known to mediate receptor trafficking to the membrane such as CXCR4, integrin β1, and CD11a [35–38]. We here demonstrate that ARF1 is also responsible for induction of functional β_2_ integrin on HSPC. We also identified a second signaling pathway, LRP1, to be involved in mediating integrin β_2_ function on HSPC. Aside from LDL uptake, LRP1 also can affect integrin expression and/or function. In macrophages, LRP1 interacts with multiple sites in α_M_β_2_, which results in integrin clustering leading to cell adhesion and migration [27,28]. LRP1 also plays a role in inducing maturation of the β1 integrin, causing a substantial increase of this receptor on myeloid cells [39]. In this study, we found that LDL induces expression of LRP1, and that deficiency of LRP1 significantly inhibits HSPC adhesion to ICAM-1 in vitro. LRP1 induction by LDL was, however, independent on pERK. Deficiency of LRP1 did not affect expression levels of β_2_ but reduced the adhesion capacity of LRP1-defieicnt LSK cells to ICAM-1. This would be consistent with the fact that LRP1 can not only cause integrin maturation and therefore expression [23] but also integrin function, as was shown in macrophages [27]. How LDL induces LRP1 expression remains unknown. Likewise, whether other forms of modified LDLs, aside from LDL isolated from plasma used in this study, influence LRP1 expression would be of interest. Finally, as LRP1 has multiple functions, we cannot exclude that other functions of LRP1 may play additional role in HSPC biology.

**Figure 7:**
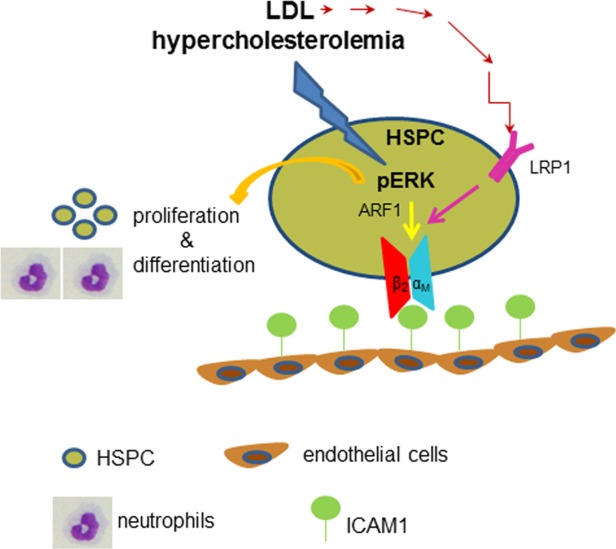
Proposed model of how hypercholesterolemia and LDL modulate HSPC homing to lesion site. Hypercholesterolemia and LDL stimulate ERK phosphorylation in HSPC. ERK activation promotes HSPC proliferation and differentiation into atherogenic myeloid cells. Apart from that, ERK activation enhances ARF1-mediated integrin β_2_ function of HSPC. In parallel, LDL increases LRP1 expression on HSPC, which in turn potentiates integrin β2 activity. As results from these changes, HSPC can attach to ICAM-1-expressing endothelial cells in lesion site where they may further contribute to inflammation and arteriosclerosis progression. Abbreviations: ARF1, ADP-ribosylation factor 1; HSPC, hematopoietic stem/progenitor cell; ICAM1, intercellular cell adhesion molecular-1; LDL, low density lipoprotein; LRP1, LDL receptor-related protein 1; pERK, phospho ERK.

Our proposed model of the molecular mechanisms underlying LDL-mediated effects on HSPC biology is depicted in Figure 7. In vivo hypercholesterolemia and in vitro exposure to LDL causes phosphorylation of ERK1/2 in LSK cells. Phospho-ERK is, as others and we previously showed, responsible for LSK proliferation and differentiation [2,3] and as shown here, adhesion, migration, and homing. Phospho-ERK induces HSPC migration via ARF1, which increases expression of functional β_2_ integrin on LSK cells. LDL also increases integrin function, not expression, on HSPC by interacting with LRP1 known to increase integrin function [27]. The proposed model extends our knowledge on the pathogenesis of hypercholesterolemia-associated arteriosclerosis in the context of HSPC, which may provide novel therapeutic targets for the treatment of arteriosclerosis.

## Summary

Hypercholesterolemia-associated leukocytosis accelerates arteriosclerosis progression. The link among HSPC proliferation and differentiation in the BM, leukocytosis, and reinforced arteriosclerosis has been noted in hypercholesterolemic mice. However, it remains undefined whether and how circulating HSPC could directly participate in arteriosclerosis. In addition, how hypercholesterolemia, specifically LDL, affects HSPC trafficking to injured arteries is largely unknown. Using LDLr^−/−^ mice model, we identified that integrin β_2_ was upregulated on HSPC of LDLr^−/−^ on HFD. Increased integrin β_2_ expression enhanced HSPC function including their adhesion and migration toward ICAM-1 and homing to injured arteries. Strikingly, grafting HSPC to injured arteries accelerated inflammation and arteriosclerosis progression. Taken together, HSPC bear proatherogenic property and could directly participate in arteriosclerosis. Hypercholesterolemia stimulates arteriosclerosis progression could be partially via its regulation of HSPC as well as mature myeloid cells.
