# Supplementary material for: Hematopoietic Stem/Progenitor Cells Directly Contribute to Arteriosclerotic Progression via Integrin β2
Source: Stem Cells. 2015 Mar 24;33(4):1230–40. doi: 10.1002/stem.1939 (PMC4409030; doi:10.1002/stem.1939)
Supplement: Supplementary file 1 [file stem0033-1230-sd1.docx]

**Supplemental data**

**Hematopoietic stem/progenitor cells directly contribute to arteriosclerotic progression via integrin α_M_β_2_**

Wang et al. Short title: Hematopoietic stem cells in arteriosclerotic plaque

**Methods**

**Mice and treatments:** Wild type C57BL/6J (CD45.2, H-2kb) mice, B.6SJL-PTPRCA (CD45.1), Balb/C Rag2- gammaC^-/-^ mice and integrin β_2_^-/-^ (i.e. CD18^-/-^) mice were used at the age of 8-12 weeks. Homozygous LDL receptor knockout (LDLr^-/-^) mice were purchased from Jackson Laboratory (Bar Harbor, Maine, U.S.A.) and backcrossed with C57BL/6J mice for at least 8 generations to achieve 98.44% C57BL/6J background. Homozygous LRP1 knock-in mutant mice (LRP1^n2/n2^) and integrin β_2_ deficient mice on a C57BL/6J background were generated as described before, respectively [[1](#_ENREF_1), [2](#_ENREF_2)]. Balb/c Rag2- gammaC^-/-^ mice (H-2kd) were purchased from Jackson Laboratory. LDLr^-/-^ mice were fed on high-fat diet (HFD) (34% fat, 1% cholesterol, Catalog no. D12492 mod, BioServices, the Netherlands) (n=89) or chow diet (n=70) for 8 weeks. Complete ligation was performed on B.6SJL-PTPRCA (CD45.1) mice, wild type C57BL/6J (CD45.2, H-2kb) mice or Balb/c Rag2- mice (H-2kd) mice for HSPC homing and injection experiment. Complete carotid artery ligation was performed in CD18^-/-^ and their littermates to assess neointima formation. Approximately in total 560 mice were included in the entire study. All mice were maintained in the animal facilities of the KU Leuven or Capital Medical University. All experiments were carried out with approval of the ethics committee of these universities.

**Lipoprotein separation by gel filtration:** Murine plasma (50 µl) was separated on fast performance liquid chromatography (FPLC) as described before [[3](#_ENREF_3)]. Cholesterol, glycerol and triglyceride content of plasma and gel filtration fractions were quantified [[3](#_ENREF_3)].

**Lipoprotein isolation by density gradient ultracentrifugation:** Plasma from human healthy volunteers was separated by density gradient ultracentrifugation. LDL (1.019 g/ml < g < 1.063 g/ml) fractions were isolated, pooled and then dialyzed in 1 mM EDTA overnight. Cholesterol level was measured as above [[3](#_ENREF_3)].

**HSPC cell isolation:** To obtain LSK cells, Lin- cells were isolated from BMCs using the Lineage cell depletion Kit (Miltenyi Biotech, Bergisch Gladbach, Germany). After they were stained with anti-lineage cocktail APC, anti-Sca-1 FITC and anti-cKit PE antibodies (Abs), LSK cells were isolated on a FACS Aria III (Becton Dickinson, NJ, U.S.A.).

**Flow cytometry:** To quantify HSPC in BM and PB, BMCs and PB cells were stained with surface markers [[3](#_ENREF_3)]. HSPC were identified as Lin- Sca-1+ cKit+ (LSK) cells and long term HSC (LT HSC) were defined as CD150+ CD48- LSK cells. To study integrin β_2_ expression on LSK cells, BMCs or cultured Lin- cells were stained with rat anti-mouse integrin β_2_ (i.e. CD18) (1 µg/1x10^6^ cells) for 20 min and then goat anti-rat Alexa 488 (1/400) for 15 min, followed by anti-Lineage cocktail APC (1/50), anti-Sca-1 PerCP-Cy5.5 (1/100) and anti-cKit APC-H7 (1/100) Abs before FACS analysis. Alternatively, cells were incubated with PE conjugated anti-mouse integrin β1, or anti-mouse integrin α5, together with anti-mouse Sca-1 FITC, anti-Lineage APC and anti-cKit APC-H7 for FACS. ERK phosphorylation in LSK cells of LDLr^-/-^ mice on chow and HFD was assessed as described before [[3](#_ENREF_3)].

FACS antibodies list:

| antibodies | clones/catalogue number | company |
| --- | --- | --- |
| anti-mouse Sca-1 PerCP-Cy5.5, & FITC | D7 | eBioscience |
| anti-mouse Sca-1 PE | D7 | BD |
| anti-mouse cKit PE | 2B8 | eBioscience |
| anti-mouse cKit APC-H7 | 2B8 | BD |
| anti-mouse lineage cocktail APC | M1/70, 145-2C11, RB6-8C5, TER-119, RA3-6B2 | BD |
| anti-mouse CD18 | M18/2 | ebioscience |
| anti-mouse CD49e | HM5-1 | eBioscience |
| anti-mouse CD29 | Ha/25 | BD |
| anti-mouse CD45.1 FITC | A20 | BD |
| anti-mouse CD45.2 PerCP-Cy5.5 | 104 | BD |
| anti-mouse CD45R/B220 APC | Ly-5 | BD |
| anti-mouse Gr-1 APC | RB6-8C5 | BD |
| Phospho-p44/42 MAPK Alexa Fluor 488 conjugated | D13.14.4E | Cell Signaling technology  (Bioké, Leiden, the Netherlands) |
| Goat anti-rat Alexa 488 | A11006 | Invitrogen |
| Streptavidin – Cy3 | 43-4315 | Invitrogen |
| Streptavidin Alexa 633 | SA1005 | Invitrogen |

All FACS analyses were performed using appropriate isotype control Abs. To obtain reliable quantification, at least 100,000 events were acquired for BMCs and 10,000 events were acquired for Lin- cells. Data were acquired using a FACS Canto (BD) or FACS Aria III (BD) apparatus.

**Cell culture:** Lin- cells were isolated as described above. Lin- cells were exposed to 0 or 100 µg/ml LDL in the presence or absence of U0126 (10 µM) for 24 hours. Cells were stained with anti Lin APC, Sca-1 FITC, cKit APC-H7 and CD49e PE, CD29 PE or CD18 to assess their expression on LSK cells, respectively.

To study the dose effects of LDL and HDL on integrin β_2_ expressing LSK cells, Lin- cells were exposed to various concentrations of LDL or HDL (0-100 µg/ml) for 24 hours. Cells were stained with anti-mouse CD18 and LSK markers for FACS analysis.

**HSPC *in vitro* adhesion assays:** To study the adhesion capacity, LSK cells were isolated by FACS and seeded at a density of 10,000 cells per well of 96-well plates pre-coated with ICAM-1 (5 µg/ml, R&D Systems). In some experiments, LSK cells were pre-incubated with anti-mouse CD18 (1 µg/1×10^6^ cells) or isotype antibody (1 µg/1×10^6^ cells) at 4°C for 20 min before seeding. Two hours after adhesion, non-adherent LSK cells were removed and adherent cells were collected after trypinization (0.25% trypsin, Invitrogen) for 2 min at 37°C. To enumerate the number of adherent cells, 50 µl counting beads with known quantity were added to each sample before FACS acquisition [[4](#_ENREF_4)]. The number of adherent LSK cells was calculated according to the following formula:

A

D

C

= concentration of cell sample (cells/µl)

×

B

Where: A = number of cell events; B = number of bead events; C = assigned bead count of the lot (beads/50 μL); D = volume of sample (μL).

**HSPC *in vitro* transmigration assay:** 3×10^5^ Lin- cells were incubated with PBS, anti-mouse CD18 (1 µg/1×10^6^ cells) or isotype antibody (1 µg/1×10^6^ cells, BD) at 4°C for 20 min before loading into the upper chamber of modified Boyden chambers (3 µm pore size, Costar, Avon, France), after coating the transwells with 5 µ/ml ICAM-1 overnight[[4](#_ENREF_4)]. After 6 hours, cells migrated into the lower chamber were collected, enumerated and stained with anti-Lineage cocktail APC, anti-Sca-1 FITC and anti-cKit PE Abs, and LSK cells were quantified by FACS. To determine the number of migrated LSK cells, the number of input LSK cells was determined by staining the input Lin- cells with the same anti-LSK Abs and the percentage of migrated LSK cells in seeded LSK cells was calculated.

**HSPC *In vivo* homing to injured artery**: To induce ICAM-1 expression, the right carotid artery of male C57BL/6 mice (n=20) was completely ligated at a position proximal to the carotid bifurcation as described before [[5-7](#_ENREF_5)]. Three days after ligation, Lin- cells were isolated by MACS and labeled with 10 µM PKH26 (Sigma-Aldrich) before injecting to the ligated mice. After 1 or 10 days, mice were euthanised and perfused thoroughly with 20-30 ml PBS via the left ventricle to remove all blood cells inside the arteries. Carotid arteries were dissected, cut into pieces (2-3 mm length) and digested with Collagenase I (3 mg/ml, R&D) and Collagenase II (1.4 mg/ml, Sigma-Aldrich) in DPBS at 37°C for 2 hours to obtain single cells [[8](#_ENREF_8)]. Cell suspensions were numerated and then stained with anti-Lineage cocktail APC, anti-cKit APC-H7 and anti-Sca-1 FITC Abs. Homed LSK cells, i.e. PKH26^+^/Lin-Sca-1^+^cKit^+^ cells or CD45.2+/Lin-Sca-1^+^cKit^+^ cells, were quantified by FACS. The absolute numbers of homed LSK cells in uninjured and injured arteries were calculated.

For inhibitor experiment, cells were incubated with anti-mouse CD18 (1 µg/1×10^6^ cells) or isotype antibodies (Abs) (1 µg/1×10^6^ cells) at 4°C for 20 min prior to injection via the tail vein (1×10^6^ cells per mouse).

**Carotid artery ligation**: The right carotid artery was complete ligated in CD18^-/-^ mice and their littermates. After 28 days of surgery, mice were dissected for H&E analysis.

**Competitive BM transplantation:** To investigate the hematopoietic potential of LSK cells that homed to the ligated carotid arteries, PKH26^+^/LSK cells were isolated by FACS. Together with 2 ×10^6^ CD45.1 BMCs, sorted PKH26^+^/LSK cells were injected into irradiated CD45.1 recipients (500 PKH26^+^/LSK cells per recipient, n=4). After 6 weeks, peripheral blood (PB) cells were harvested, red blood cells were lysed, and white blood cells were stained with anti-CD45.1 FITC, anti-CD11b PE, anti-CD45.2 PerCP-Cy5.5 and anti-Gr-1 APC Abs, or isotype control Abs to study myeloid lineage reconstitution by FACS. In parallel, PB cells were stained with anti-CD45.1 FITC, anti-CD45.2 PerCP-Cy5.5 and anti-B220 APC or isotype control Abs to evaluate B cell reconstitution.

**HSPC *in vivo* injection to mice with injured artery:** Balb/c Rag2- gammaC^-/-^ mice (H-2kd) were performed a complete ligation on right carotid artery. To enhance homing efficiency, splenectomy was carried out 3 days after ligation. Seven days after ligation, 40,000 CD18^+/+^ or CD18^-/-^ LSK cells (H-2kb) were sorted out by FACS and administered to ligated Balb/c Rag2- gammaC^-/-^ mice via tail vein. Recipients were dissected 28 days after ligation.

**Histology:** When dissection, mice were perfused with saline and then 4% paraformaldehyde. Hearts or ligated arteries were dissected. Cryosections of 7 µm thickness were obtained. Morphometric analysis was performed on H&E-stained slides using KS300 software (Carl Zeiss, Oberkochen, Germany).

**Immunohistochemistry:** Cryosections of ligated arteries were probed with rat anti-mouse CD45 (5 µg/ml, BD Pharmingen) and mouse anti-mouse H-2kb (5 µg/ml, Clone AF6-885, BD Pharmingen) overnight and then incubated with goat anti-rat Alexa 488 1/500 and goat anti-mouse Alexa 555 1/500. Inflammatory cells were analyzed as mentioned above. For negative controls, slides were probed with rat anti-mouse IgG (5 µg/ml, DAKO) and isotype PE (1/20, BD Biosciences) overnight and then labeled with goat anti-rat Alexa 488 1/500 and goat anti-mouse Alexa 555 633 1/500. HSPC in the atherosclerotic plaques were identified and quantified using the Axiovision software (Axioimager Z1 microscope, Carl Zeiss) on Z-Stack images after extended focus computation (Carl Zeiss).

**Western blot:** Protein lysates were generated from Lin- cells from C57BL/6 mice treated with or without 100 µg/ml LDL and with or without 10 µM U0126 for 24 hours using RIPA. Protein lysates (30 µg per lane) were separated on 4-20% gradient SDS-PAGE gels (Bio-Rad, CA, U.S.A.). After transfer, membranes were probed with murine anti-ARF1 1/50 (AssayDesigns, Inc., Ann Arbor, MI), rabbit anti-mouse LDLr 1/500 (Cell Signaling, Bioké, Leiden, the Netherlands), rabbit anti-mouse LRP1 1/2000[[1](#_ENREF_1)], or rabbit anti-mouse β-actin 1/2000 (Cell Signaling) Abs overnight. Membranes were then probed with the appropriate secondary Abs coupled to horseradish peroxidase. Signals were detected by ECL chemiluminescence (Thermo Fisher, Scientific, Rockford, IL, U.S.A.).

**Statistics**: Data were expressed as mean ± SEM. In BFA inhibition experiments, paired, 2-tailed Student’s t test was used to compare control and BFA. In all other experiments where there were 2 experimental groups, unpaired, 2-tailed Student’s t test was used for data with normal distribution. For data that did not fit normal distribution, nonparametric Mann Whitney analysis was used. One-way ANOVA with Dunnett was used when comparing treated groups against control and ANOVA with Bonferroni was applied when comparing all groups. Correlation was performed with Pearson analysis. Statistics analysis was performed using GraphPad Prism (GraphPad Software Inc, La Jolla, CA, U.S.A.). A *P* value less than 0.05 was considered significant.

**Supplemental figures and figure legends**

**Supplementary figure 1**. **High fat diet induced hyperlipidemia in LDLr^-/-^ mice.** At the age of 8-12 weeks, LDLr^-/-^ mice were fed on chow or high fat diet (1% cholesterol, 34% fat) for 8 weeks. When dissection, mice were fast overnight and plasma was collected. (A) Total cholesterol, total glycerol, free glycerol and triglyceride (TAG) were determined. n=5. (B) To study the effect of diet on lipoproteins, 50 µl plasma was separated by gel filtration. Representative FPLC chromatography was shown in which VLDL/LDL and HDL were indicated.

**Supplementary figure 2**. **Kinetics of donor-derived LSK cells homing to carotid arteries.** Right carotid artery (RCA) of CD45.1 mice was ligated completely as described before. Seven days after ligation, Lin- cells were isolated from LDLr^-/-^ mice on high fat diet and injected to ligated CD45.1 recipients via tail vein. At day 1 and day 10 after cell injection, both carotid arteries were dissected from ligated CD45.1 mice, digested into single cells and numerated. Cells were stained with anti-mouse CD45.2 and LSK markers for FACS. The percentage of CD45.2+ LSK cells was obtained to calculate the absolute number of homed LSK cells. LCA: left carotid artery; RCA: right carotid artery.

** Supplementary figure 3**. **Dose effects of LDL and HDL on integrin β_2_ expressing LSK cells in vitro.** Lin- cells were isolated from LDLr-/- mice on chow diet. They were cultured in various concentrations of LDL (0-100 µg/ml) and HDL (0-100 µg/ml) for 24 hours. Cells were stained with anti-mouse CD18 PE, anti-mouse cKit FITC, anti-mouse Lineage APC and anti-mouse Sca-1 PerCP-Cy5.5 for FACS. The percentage of CD18+ LSK cells in LSK population was obtained. Data are expressed as fold change when compared to non-treated cells. * *P*<0.05 when compared to 0 µg/ml LDL; # *P*<0.05 when compared to 100 µg/ml HDL.

**References:**

1. Gordts PL, Reekmans S, Lauwers A et al. Inactivation of the LRP1 intracellular NPxYxxL motif in LDLR-deficient mice enhances postprandial dyslipidemia and atherosclerosis. **Arterioscler Thromb Vasc Biol***.* 2009;29:1258-1264.

2. Oreshkova T, Wang H, Seier AM et al. Beta(2) integrin deficiency yields unconventional double-negative T cells distinct from mature classical natural killer T cells in mice. **Immunology***.* 2009;128:271-286.

3. Feng Y, Schouteden S, Geenens R et al. Hematopoietic stem/progenitor cell proliferation and differentiation is differentially regulated by high-density and low-density lipoproteins in mice. **PLoS One***.* 2012;7:e47286.

4. Christensen JL, Wright DE, Wagers AJ et al. Circulation and chemotaxis of fetal hematopoietic stem cells. **PLoS Biol***.* 2004;2:E75.

5. McPherson JA, Barringhaus KG, Bishop GG et al. Adenosine A(2A) receptor stimulation reduces inflammation and neointimal growth in a murine carotid ligation model. **Arterioscler Thromb Vasc Biol***.* 2001;21:791-796.

6. Noma K, Rikitake Y, Oyama N et al. ROCK1 mediates leukocyte recruitment and neointima formation following vascular injury. **J Clin Invest***.* 2008;118:1632-1644.

7. Squadrito F, Deodato B, Bova A et al. Crucial role of nuclear factor-kappaB in neointimal hyperplasia of the mouse carotid artery after interruption of blood flow. **Atherosclerosis***.* 2003;166:233-242.

8. Psaltis PJ, Harbuzariu A, Delacroix S et al. Identification of a monocyte-predisposed hierarchy of hematopoietic progenitor cells in the adventitia of postnatal murine aorta. **Circulation***.* 2012;125:592-603.
